# Boron Deficiency Effects on Sugar, Ionome, and Phytohormone Profiles of Vascular and Non-Vascular Leaf Tissues of Common Plantain (*Plantago major* L.)

**DOI:** 10.3390/ijms20163882

**Published:** 2019-08-09

**Authors:** Benjamin Pommerrenig, Kai Eggert, Gerd P. Bienert

**Affiliations:** 1 Department of Physiology and Cell Biology, Leibniz Institute of Plant Genetics and Crop Plant Research (IPK), Corrensstraße 3, D-06466 Gatersleben, Germany; 2 Plant Physiology, University of Kaiserslautern, Paul-Ehrlich-Str. 22, D-67653 Kaiserslautern, Germany

**Keywords:** boron, vasculature, phloem, flowering, cytokinin, brassinosteroid, abscisic acid, nutritional status

## Abstract

Vascular tissues essentially regulate water, nutrient, photo-assimilate, and phytohormone logistics throughout the plant body. Boron (B) is crucial for the development of the vascular tissue in many dicotyledonous plant taxa and B deficiency particularly affects the integrity of phloem and xylem vessels, and, therefore, functionality of long-distance transport. We hypothesize that changes in the plants’ B nutritional status evoke differential responses of the vasculature and the mesophyll. However, direct analyses of the vasculature in response to B deficiency are lacking, due to the experimental inaccessibility of this tissue. Here, we generated biochemical and physiological understanding of B deficiency response reactions in common plantain (*Plantago major* L.), from which pure and intact vascular bundles can be extracted. Low soil B concentrations affected quantitative distribution patterns of various phytohormones, sugars and macro-, and micronutrients in a tissue-specific manner. Vascular sucrose levels dropped, and sucrose loading into the phloem was reduced under low B supply. Phytohormones responded selectively to B deprivation. While concentrations of abscisic acid and salicylic acid decreased at low B supply, cytokinins and brassinosteroids increased in the vasculature and the mesophyll, respectively. Our results highlight the biological necessity to analyze nutrient deficiency responses in a tissue- rather organ-specific manner.

## 1. Introduction

Boron (B) is an essential micronutrient for seed plant species. Boron serves as a structural element in the cell wall’s pectin layer where boric acid links two rhamnogalacturonan II (RG-II) monomers via di-ester bonds with RG-II intrinsic apiose sugar residues. Consistently, B deficiency leads to instability of cell walls, associated with root and shoot meristematic defects and male sterility of floral organs [1,2].

The substrates for B-transporting proteins in plants are the molecules boric acid (H_3_BO_3_) and borate (B(OH_4_^−^) [3]. Because of the limited solubility of the latter, and bad remobilization efficiency of B after incorporation into cell walls, a continuous supply of B during a plants’ life cycle is crucial for proper development and fertility. B is taken up by the roots and transported to shoots. The uptake of B into root cells and its subsequent export into xylem vessels are driven by the interplay of passive boric acid channels of the Nodulin 26-like intrinsic protein family (NIPs) and secondary active borate transporters, the so-called BORs [4].

The damaging impact of B deficiency on the plant vasculature was reported [5,6], and veins from B-deprived leaves often adopt a serpentine shape-like growth behavior leading to the reduced longitudinal growth of leaf blades and cell expansion within the leaf mesophyll [1,7]. Such phenotypes can in part be explained by the lack of stability of cell walls in B-deprived organs leading to a collapse of cell layers and transport vessels. Interestingly, amongst plant species with very high B demands, varieties exist, which can maintain leaf and vascular growth even under highly unfavorable B starvation conditions and under a very low B nutritional tissue status [7,8]. Boron deficiency tolerance mechanisms in such B-undemanding varieties may include a differential regulation of B-sensing and response triggering networks. As recently reported for rapeseed, plant hormones are most likely important players in primary B sensing cascades and B deficiency response reactions [9,10]. Both the perception and signaling of the B nutritional status and the B-dependent plant hormone metabolism must be logistically and structurally linked to the information-conducting vascular tissues. However, direct molecular analyses of vascular tissues under B-deficient conditions are lacking to date. The present study, therefore, not only aimed to gain information about vascular bundle- and mesophyll-specific distribution patterns of sugars, nutrients, and phytohormones, but also to compare these patterns between B-deficient and B-sufficient growth conditions.

Leaf vascular tissue is experimentally not readily accessible for analysis of gene expression and metabolites in most plant taxa and extraction of vascular and remaining leaf tissue requires either mechanical [11] or proteolytic [12] separation techniques which are invasive or lengthy in nature and may, thus, alter the original profile of the substances to be analyzed. Vascular bundles of species from the *Plantago* genus are surrounded by a mechanically stable endodermis which forms Casparian strips [13], typically found only in the root endodermis of plants. This architectural peculiarity renders *Plantago* vasculature extremely stable and adds protection to fragile phloem elements. Many *Plantago* species demonstrate high tolerance against multiple abiotic stresses. For instance, this special vascular bundle anatomy enables *Plantago* leaves to withstand harsh environmental conditions by efficiently transporting water and solutes even under severe salt or osmotic stress or under heavily mechanical stresses like trampling [14,15,16,17].

The unique architecture of the vasculature of *Plantago* also permits easy experimental accessibility and ‘contamination-free’ separation from surrounding mesophyll tissues. Unlike to other plant species, large quantities of pure and intact vascular bundles can be manually pulled out of leaves and petioles from common plantain (*Plantago major* L.) and separated within a comparably short time (less than 20 s per preparation) leaving hardly time for wound-induced synthesis of stress compounds. During manual extraction of the vascular bundles, surrounding mesophyll tissue cells detach from the endodermal vascular border and isolated vascular strands from common plantain are, therefore, almost free from cellular mesophyll contaminations [13]. Such isolated vascular (and the remaining leaf) tissue were used for vascular- versus non-vascular-specific analyses of physiological parameters [18], gene expression and transcriptomics [19,20,21], and secondary metabolites [22,23]. As noted recently, this technique leaves the smallest veins still embedded within the surrounding mesophyll tissue of petioles and leaves [21], and the obtained vascular fraction might, therefore, be over-representative for transport rather than for phloem loading moieties.

In this study, we determined vascular and mesophyll sugar, nutrient, and plant hormone profiles in *P. major* and combined the accessibility and separation purity of both tissues with the possibility to use a soil substrate-based cultivation system, which allows plants to grow in a defined B supply regime. Recently, we set up this cultivation system for the identification and analysis of rapeseed cultivars with different B efficiencies [7]. The combination of the particular cultivation system and experimentally valuable quality of *P. major* allows us to dissect the impact of different B conditions on vascular and mesophyll biology. The usage of the by us developed soil substrate cultivation system allows for a more natural growth analysis than, e.g., hydroponic culture growth assays in which a physiologically and plant nutritionally-relevant rhizosphere is absent.

## 2. Results and Discussion

### 2.1. Low B Conditions Do Not Affect Vegetative but Generative Growth of P. major

*P. major* plants were germinated and grown under two different soil conditions, namely a B-sufficient (= B1 = 2.4 mg B (kg soil substrate)^−1^), and a presumably B-limiting condition (= B0 = 0.1 mg B (kg soil substrate)^−1^). A special soil substrate with extremely low nutritional value, referred to as “zero soil” containing a very high proportion of peat was used for these analyses [9]. This soil substrate and these plant growth conditions were recently applied in a comprehensive screening approach for B-efficient rapeseed (*Brassica napus* L.) genotypes [7]. There, the limiting B0 condition strongly affected the growth and development of most of the analyzed genotypes, demonstrating that plants were indeed inadequately supplied with B from the soil reservoir. Only three of about 590 genotypes exhibited similar growth rates under B1 and B0, classifying these genotypes as highly B-efficient [7].

**Figure 1:**
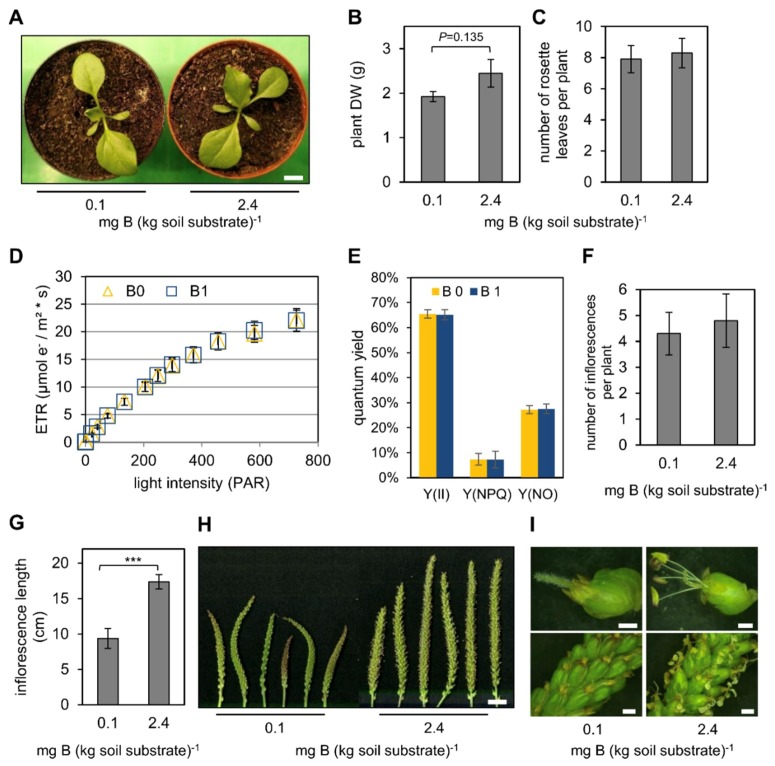
Growth parameters of *P. major* and pulse amplitude modulated (PAM) fluorometry data. (**A**) Representative pictures of four-week-old *P. major* plants grown under low B (= B0 = 0.1 mg B (kg soil substrate)^−1^) or normal B conditions (= B1 = 2.4 mg B (kg soil substrate)^−1^). (**B**) Dry weight and (**C**) number of rosette leaves of plants grown at 0.1 or 2.4 mg B (kg soil substrate)^−1^. Bars are means from *n* = 12 plants ± SD. *p-*value was determined by double-sided *t*-test. (**D**) Electron transfer rate (ETR) measured on whole rosettes in dependence of light intensity (PAR). Values are means of *n* = 4 plants per condition ± SE. (**E**) PAM fluorometry parameters. Quantum yields of photosystem II activity [Y(II)], non-photochemical quenching [Y(NPQ)], and non-regulated electron transfer [Y(NO)] are depicted. Bars are means of *n* = 4 plants ± SE. (**F**) Number of inflorescences per plant and (**G**) average length of inflorescences from plants grown at 0.1 or 2.4 mg B (kg soil substrate)^−1^. Values are means from *n* = 12 plants ± SD. Asterisks represent *p*-value < 0.001 according to double-sided *t*-test. (**H**) Representative inflorescences from plants grown at 0.1 or 2.4 mg B (kg soil substrate)^−1^. White scale bar is 1.8 cm. (**I**) Representative pictures from flowers (upper two pictures) and magnified inflorescences (lower two pictures) from plants grown at 0.1 or 2.4 mg B (kg soil substrate)^−1^. White scale bars are 4 mm (flowers) and 25 mm (inflorescences).

When *P. major* plants were grown on the aforementioned soil substrate, no apparent differences between B0 and B1-grown plants were observed during vegetative growth (Figure 1A). Plant biomass, measured as shoot dry weight (DW) of 6-week-old plants, was lower under B0 than under B1 conditions; however, the likelihood for this difference was only 86.5% (double-sided *t*-test, *p* = 0.135) (Figure 1B). Moreover, the number of rosette leaves of these plants did not differ between B0 and B1 (Figure 1C) indicating that B limitation did not affect vegetative growth in a similar manner as recently observed for *B. napus* and *Arabidopsis thaliana* (L.) Heynh. [7,10]. To reveal possible effects of B nutrition on photosynthetic efficiency, photosynthetic parameters were determined. Electron transfer rates and photosynthetic quantum yields were similar between B0 and B1-grown plants (Figure 1D,E). The relative proportions of the quantum yield between photosystem II activity [Y(II)], non-photochemical quenching [Y(NPQ)], and unregulated energy dissipation [Y(NO)] were unaltered between the contrasting B conditions indicating that photosynthetic electron transport was not affected in a B-dependent manner (Figure 1E). In contrast to the undiscriminating vegetative growth behavior, obvious morphological differences appeared when plants developed inflorescences. Although the number of inflorescences per plant did not differ between the contrasting soil B conditions (Figure 1F), they were significantly shorter under B0 compared to B1 (Figure 1G,H). In particular, male flower parts did not develop properly in B0 conditions leading to sterility and inability of the plants to form seeds (Figure 1I). In summary, these results showed that B is crucial for proper pollen formation, but apparently dispensable during vegetative growth.

### 2.2. Low B Conditions Affect Vascular Sucrose Accumulation and Sucrose Phloem Loading

Sugar alcohols like sorbitol and inositol can form B esters, and the existence of B-polyol complexes in polyol-translocating species was reported [24,25]. *P. major* translocates both, sorbitol and sucrose in the phloem in very high concentrations (up to 300 mM sorbitol and 600 mM sucrose) [20,26]. We, therefore, analyzed whether B nutrition would affect the accumulation of those translocating sugars along with the monosaccharides and sorbitol precursors glucose and fructose. 

**Figure 2:**
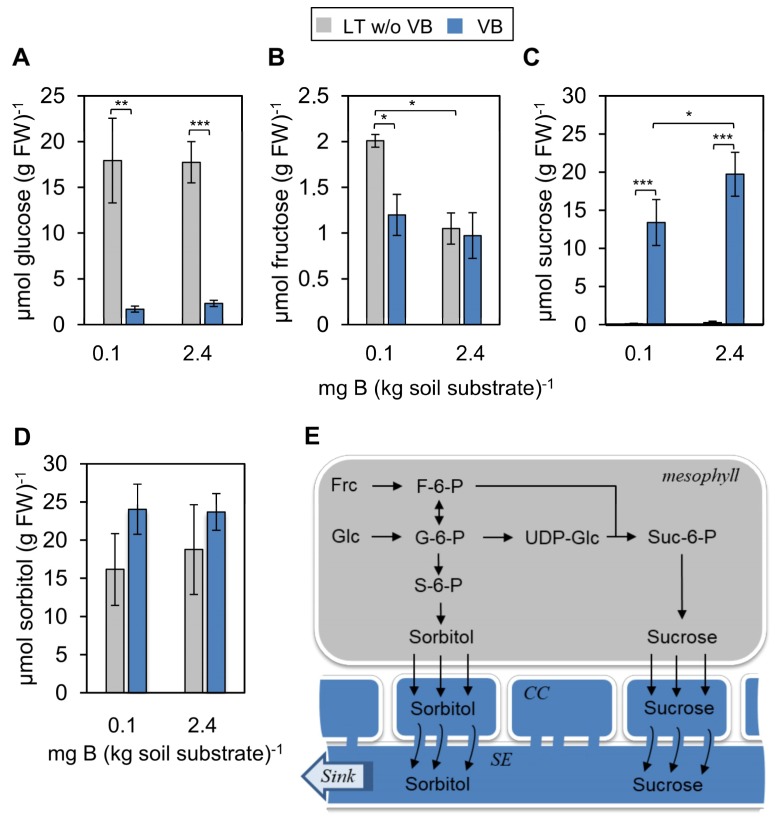
Quantification of sugars in vascular bundles (VB; blue bar charts) and leaf tissue without vascular bundles (LT w/o VB; grey bar charts) in *P. major* plants grown on two different soil B conditions. (**A**) The concentration of glucose, (**B**) fructose, (**C**) sucrose, and (**D**) the sugar alcohol sorbitol. Bars represent means of at least *n* = 6 samples ± SD. Asterisks represent *p*-values < 0.05 (*), <0.01 (**), <0.001 (***) according to double-sided *t*-test. (**E**) Schematic depiction of *P. major* phloem loading and the biosynthesis of the analyzed sugars. Sugars are synthesized in the mesophyll tissue and the transport forms of sugars, which are sorbitol and sucrose, are loaded via specific transporters into companion cells (CC) from where they travel symplasmically into phloem sieve elements (SE) and along with the phloem stream into sink organs. Abbreviations: Frc = fructose, Glc = glucose, G-6-P = Glc-6-Phosphate, F-6-P = Frc-6-Phosphate, S-6-P = Sorbitol-6-Phosphate, Suc-6-P = Sucrose-6-Phosphate).

Concentrations of individual sugars were different between vascular bundles and mesophyll and clearly reflected the different physiological and biochemical processes within these tissues (Figure 2). Whereas glucose levels were high in the leaf fraction without vascular bundles and low in vascular bundles, sucrose concentrations were about 100-fold higher in the isolated bundle fraction in comparison to the remaining leaf tissue (Figure 2). These differences demonstrate the function of glucose as primary sugar product of the Calvin-Benson cycle in the photosynthetically active leaf tissue and that of sucrose as the main transport form of carbon via the phloem stream in the vascular bundles. These tissue-dependent differences in the sugar concentrations also clearly proofed the purity of the individually obtained separated tissues, that are vascular bundles and mesophyll.

The quantification of the soluble sugars did not reveal any significant differences in glucose and sorbitol concentrations between the two contrasting soil B conditions (Figure 2A,D). However, quantitative differences were observed for fructose and sucrose concentrations (Figure 2B,C). Fructose levels significantly increased, specifically, in the mesophyll of plants grown under the B0 condition (Figure 2B). Sucrose concentrations, on the other hand, decreased by about 25% in the vascular bundles (Figure 2C). This high reduction in concentration suggested that sucrose was either less synthesized or loaded at lower efficiency into the phloem in plants grown under low B.

**Figure 3:**
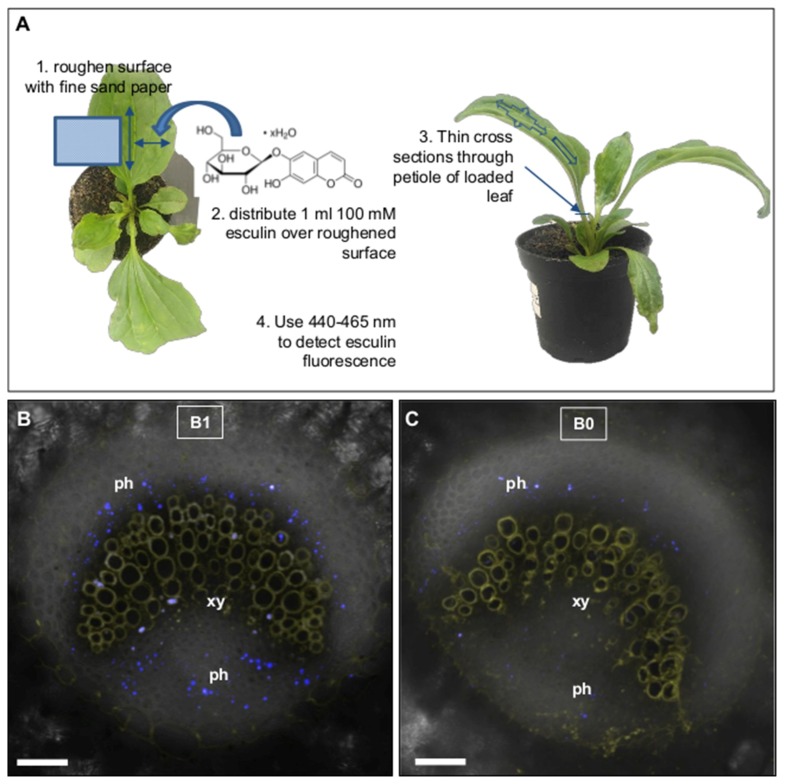
Distribution of esculin in the phloem of *P. major* grown under different soil B conditions. (**A**) schematic representation of the experimental set-up. (**B,C**) Representative cross-sections of vascular bundles from petioles. Yellow fluorescence indicates lignified xylem structures, and blue spots represent esculin-derived fluorescence in the phloem 3 h after loading of 100 mM esculin solution onto the adaxial side of source leaves of *P. major* plants grown under normal (**B**) (= B1 = 2.4 mg (kg soil substrate)^−1^) or low B (**C**) (= B0 = 0.1 mg B (kg soil substrate)^−1^) conditions. Source leaves of 10 plants per condition were treated with esculin and showed similar results. Abbreviations: ph = phloem, xy = xylem. Bars are 50 µm.

To reveal whether the lower vascular sucrose concentration at B0 compared to B1 was due to reduced loading of assimilates into the phloem, we made use of the fluorescent sucrose analogue esculin. The coumarin glycoside esculin is phloem mobile [27] and recognized specifically by type II SUT/SUC sucrose transporters of dicotyledonous plant species [28,29,30], which are responsible for apoplastic sucrose phloem loading [31,32,33] also in *P. major* [34]. To analyze esculin uptake and trafficking into and within the phloem, an excess of esculin was applied in solution onto the roughened adaxial surface of fully developed source leaves, and esculin-derived fluorescence was tracked 3 to 5 h after loading in the phloem of petioles of treated leaves (Figure 3). Confocal analysis of UV-excited cross-sections through petioles revealed the bi-collateral architecture of *P. major* [13,34], i.e., the crescent-shaped xylem (emitting fluorescence in the yellow range of visible light) being flanked on the adaxial and abaxial sides with phloem cells, represented by the blue dots of esculin-derived blue fluorescence (Figure 3A,B). Interestingly, the number of blue fluorescing cells was much lower in B0 in comparison to B1 plants (Figure 3). Since esculin was applied in great excess on the leaf surfaces—and, therefore, not limiting to phloem loading proteins—of both B0 and B1 grown plants, this result showed that esculin was loaded to a lesser extent into the phloem of B0 in comparison to B1 grown plants and indicated that also loading of sucrose was affected in the same manner. This result also supported the lower sucrose concentration measured in the isolated vascular bundles from B0 in comparison to B1 grown plants (Figure 2D). The reduction of vascular bundle-transported sucrose might, in part, explain the shorter inflorescences and impaired floral development (Figure 1). Although the stem possesses photosynthetically active cells and is able to produce a certain amount of sugars by itself, the developing flowers most likely represent strong sinks for both nutrients and building blocks. Under B limiting conditions reduced phloem sugar concentrations might then not sufficiently fuel proper growth and development of the flowers, and, therefore, be *inter alia* causative for the phenotype.

The so far obtained results strongly suggest that despite an unaltered morphology of leaves and photosynthetic activity during vegetative growth, vascular bundle-related metabolism was affected to a certain extent by the low B condition. Therefore, the ionome and plant hormone (PH) profiles in enriched vascular bundles and mesophyll fractions without vascular bundles were separately analyzed in the following. The functionality of the vasculature, in particular, the xylem and phloem, is crucial for uptake, distribution, and remobilization of nutrients. Moreover, proper differentiation of vascular and mesophyll tissue depends also to a large extent on PH-triggered developmental stimuli.

### 2.3. Low B Growth Conditions Cause Qualitative and Quantitative Changes in Nutrient Levels both in Vascular and Non-Vascular Tissues

**Figure 4:**
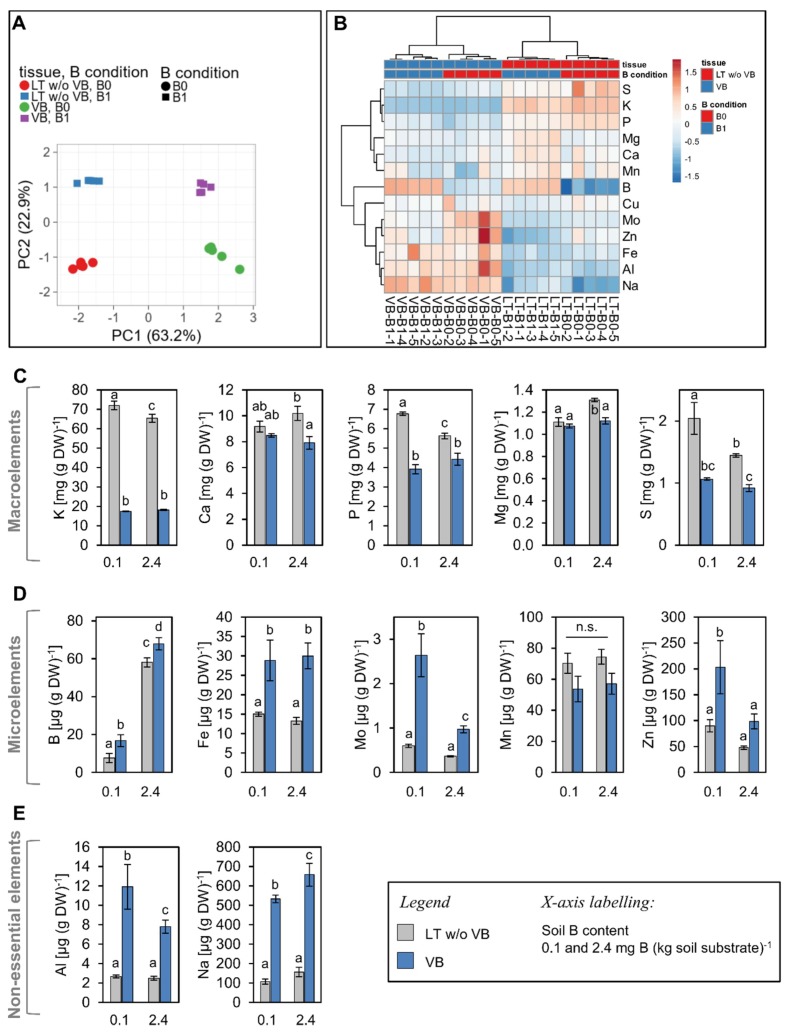
Concentrations of nutrients and mineral elements in vascular bundles (VB) and leaf tissue without vascular bundles (LT w/o VB) from *P. major* plants grown under 0.1 (B0) or 2.4 mg (B1) B (kg soil substrate)^−1^. (**A**) Principal component (PC) analysis (PC1 *versus* PC2) of the different tissues and B conditions based on elemental concentrations. Original values are ln(x + 1)-transformed. Pareto scaling was applied to values. Information about PCA loadings and explained variances of PCs can be found in Supplemental Figure S1 (**B**) Hierarchical cluster analysis of nutrient distributions determined in five pools à six plants (1–5). Results are represented by a dendrogram and heatmap showing relationships of nutrients and samples. Both rows and columns are clustered using correlation distance and average linkage. (**C**) Concentrations of macroelements, (**D**) concentrations of microelements, (**E**) concentrations of non-essential elements. (**C–E**) Bars represent mean values of indicated element concentration ± SD, *n* = 5 (DW = dry weight). Error bar labels with different letters indicate significant differences at *p* < 0.05 between treatments and tissues according to One-way ANOVA with post-hoc Tukey test. n.s. = no significant differences (*p* > 0.05).

In general, most nutrients differentially concentrate within vascular bundles or leaf tissue without vascular bundles (Figure 4). Principal component (PC) and heat map analysis, performed on the elemental concentration values of tissue samples revealed tissue- and B treatment-dependent differences (Figure 4A,B). PC1 explained 63.2% and PC2 22.9% of the total variance of the elemental concentrations between the two different tissues and B treatments. Both tissues and B treatments separated, clearly, from each other, indicating that the vascular and mesophyll nutriome responded differently to the low B treatment (Figure 4A). The heat map representation shows that concentrations of macronutrients were higher and that of most micronutrients lower in the mesophyll in comparison to the vascular tissue under normal B supply (Figure 4B). Under low B, some of these nutrients exhibited different tissue-specific accumulation. In the following, we will focus on the discussion of elements, whose levels responded to the low B condition.

#### 2.3.1. Boron

In plants growing under the B0 condition, B concentrations were reduced to 25% and 13% of the B1 value in the VB and LT w/o VB, respectively (Figure 4D, first panel). Interestingly, the sorbitol translocating *P. major* plants managed to maintain almost twice as much B (in means of percentage of B1 values, as well as in absolute values) in the vascular tissue in comparison to the remaining leaf tissue under the low B condition (Figure 4D). It has been discussed that B forms ester bonds with sugar alcohols *in planta* [24,25]. It is possible that the *P. major* phloem sap, which contains high sorbitol concentrations [20,26] acts as a dynamic B reservoir allowing accumulation and transfer of B even under soil B conditions that are insufficient for growth and stem elongation of plant species like *B. napus* or *A. thaliana* [7,35]. Correspondingly, high sorbitol mesophyll concentrations (Figure 2A) may contribute to the formation of additional sorbitol-B complexes in the remaining leaf tissue. While these concentrations were sufficient to sustain a normal development of vegetative plant parts, apparently, they were not sufficient for proper flower development, a process known to have an increased B demand (Figure 1). Our study highlights, together with other recent studies [25], the need to investigate in more detail the role of the vasculature on B logistics in different plant species.

Interestingly, B-deficient growth conditions did not decrease the level of many other quantified elements neither in the VB nor the LT w/o VB except for sodium (Na) and magnesium (Mg) in the vascular and mesophyll tissue, respectively. B, Mg and Na were the only elements which significantly decreased under B limiting growth conditions (Figure 4). It has been reported, that plants compensate for the lack of B in the cell wall with an increased accumulation of Ca, another structurally important cell wall element. However, increased Ca levels were not detected by us in *P. major* plants gown under B0 growth conditions (Figure 4C).

#### 2.3.2. Potassium and Sodium

The most striking tissue-dependent differences were observed for potassium (K) and Na, which accumulated reciprocally predominantly in the mesophyll or vasculature, respectively and whose levels were additionally affected by the soil B conditions. Notably, K was 3.6-fold higher concentrated in the mesophyll than in the vascular bundles, and Na concentrations were 4.2-fold higher in the vasculature than in the mesophyll. This represents the function of K as the main electrolyte in mesophyll cells, where K adjusts cytosolic osmolarity [36]. In vascular bundles, where K is present at lower amounts, osmolarity control is rather mediated by osmotically active soluble sugars, mainly in the form of sucrose. The high compartmentalization of Na in the vasculature, on the other hand, is interesting and may be part of Na exclusion strategy of the genus *Plantago*. Long-distance transport mechanisms for Na have been reported in *A. thaliana* [37] and *Plantago maritima* L., a salt-tolerant relative of *P. major* [26,38]. There, Na transport occurs mainly by acidification of the xylem sap, indicative for the involvement of active proton-coupled Na transport mechanisms in this tissue [38]. In our growth experiments, Na levels in the vasculature decreased by almost 20% under the low B condition, while K levels remained unchanged. However, K concentrations slightly increased in the mesophyll (Figure 4C). B-dependent changes of Na levels have been observed in rapeseed before, where Na decreased in seedlings and developing seeds [9], but increased in green tissues of adult plants under B deficiency [7]. Na^+^ -coupled borate [B(OH)_4_^−^] transport possibly catalyzed by plant BOR transporters, as described for the human B(OH)_4_^−^ transporter NaBC1 [39], may explain such observed linked fluctuations of B and Na; however, transport mechanistic of the plant BOR transporters (e.g., proton- or sodium driven) have not been conclusively revealed.

#### 2.3.3. Sulphur

The higher sulfur (S) concentration in the mesophyll in comparison to the vasculature is surprising, since many S-containing compounds and their biosynthesis, e.g., glucosinolates, thioredoxin, metallothionein have been reported to accumulate to large amounts in the vasculature, mainly the phloem [19,40,41]. In this study, however, S increased markedly under low B, but mainly in the leaf tissue without vascular bundles (Figure 4C). The only known, however, weak link between B and S metabolism is the biosynthesis of polyamines. This is because B deficiency induces the biosynthesis of stress-related polycationic polyamines putrescine and spermidine and formation of polyamine-polyphenol conjugates [42,43]. Biosynthesis of polyamines occurs in an S-dependent manner [44], and B-deprived plants may have higher S demands than control plants because of increased polyamine biosynthesis. Nevertheless, polyamines and polyamine biosynthesis in *P. major* and *A. thaliana* have been shown to be particularly specific for the vascular tissue [23], and the observed increase of S in the mesophyll cannot be fully explained by an increased demand of these compounds. 

#### 2.3.4. Metals: Aluminum, Iron, Molybdenum, Zinc

Iron (Fe) was present at 2.3-fold higher concentrations in the vascular tissue in comparison to the remaining leaf tissue; however, Fe concentrations did not change under low B conditions (Figure 4D). Two other micronutrients, namely molybdenum (Mo) and zinc (Zn) had higher concentrations in vascular bundles under low B conditions than under sufficient B (Figure 4D). Also, the toxic metal aluminum (Al) accumulated to significantly higher levels in the vasculature of plants, which grew under low B when compared to high B (Figure 4E). The increased levels of Al, but also of Mo and Zn under low B suggest either an increased uptake and translocation of these metals via low B-induced transport mechanisms into the xylem or phloem or an increased root cell wall Al affinity under B-deficient conditions. For Al, it has been reported that B can alleviate its toxicity by promoting ascorbate- and ROS defense-related factors [45,46] and by reducing Al uptake from the soil [46]. Moreover, B deficiency results in an increased number of binding sites for Al [47], which explain the increased Al levels under low B conditions as observed here.

B and Mo contents of plants seem to be linked via the activity of the enzyme nitrate reductase (NR), for which Mo is an essential cofactor. B deficiency, as well as toxicity, affect nitrate levels in a significant manner in different species leading to nitrate and proline accumulation under B deficiency [48,49]. Reduced NR activity and elevated nitrate levels may then signal elevated Mo cofactor demand and uptake from the soil. 

Moreover, it has been shown that high concentrations of Al and Zn, but also of toxic nickel and cobalt induce callose formation in the phloem and reduce loading of sucrose into minor veins [50,51]. A similar scenario cannot be conclusively transferred to the circumstances observed here, however, it might be speculated that increased concentrations of these metals in the vasculature under low B already induced countermeasures at the cell and tissue levels, namely callose phloem deposition, which may contribute to the observed reduced vascular sucrose concentration and reduced loading of esculin into the phloem (Figure 2 and Figure 3). 

### 2.4. Boron Deficiency Alters Plant Phytohormone (PH) Profiles of Vascular and Mesophyll Tissue

In the last decade, a few studies addressed the possibility that perception of B deficiency might be transmitted to B-related response networks by specific PH [5,10,52,53,54]. These studies identified possible roles in the B deficiency response for, e.g., ethylene and cytokinins in the roots [54,55] or brassinosteroids in the shoots [52]. The studies were conducted with either *A. thaliana* [54], *Pisum sativum* L. [53], or *B. napus* [10,52], species which offer very limited experimental accessibility to their vascular systems. Although it became clear through recent years that different plant hormones act differently on the development and stress responses of the vasculature and the mesophyll [56,57] and that B deficiency had specific effects on growth and development of the plant vasculature [6,7], no direct B deficiency-related PH analyses have—at least to our knowledge—been conducted of vascular tissue. Using *P. major* as a model plant system, we are able to exploit an additional level of complexity of the regulation of the B nutritional status via the phytohormone network by dissecting tissue-specific hormonal responses in vascular and mesophyll tissue.

#### 2.4.1. Cytokinins (CKs)

**Figure 5:**
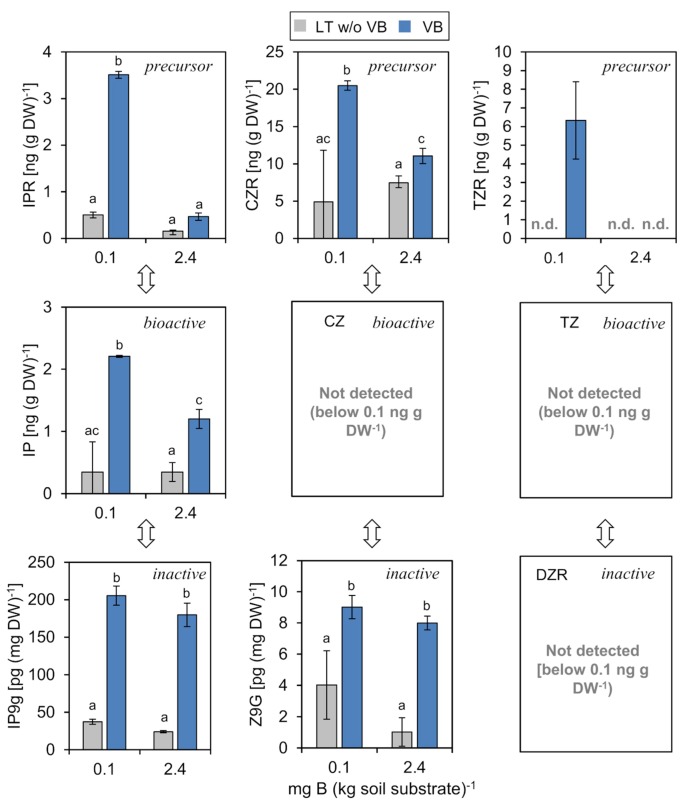
Concentrations of precursor, bioactive, and inactive cytokinin species in separated vascular bundles (VB) or leaf tissue without VB (LT w/o VB) of *P. major* plants grown in B-deficient (0.1 mg B (kg soil substrate)^−1^) or B-sufficient (2.4 mg B (kg soil substrate)^−1^) soil conditions. Arrows indicate biosynthetic conversions of the depicted cytokinin species. Bars represent averages from three different measurements ± SD. Abbreviations: DW = dry weight, IPR = Isopentenyladenine-riboside, CZR = *cis*-zeatin-riboside, TZR = *trans*-zeatin-riboside, IP = Isopentenyladenine, CZ = *cis*-zeatin, TZ = *trans*-zeatin, IP9g = Isopentenyladenine *N9*-glucoside, Z9G = Zeatin *N9*-glucoside, n.d. = not detected. Error bar labels with different letters indicate significant differences at *p* < 0.05 between treatments and tissues according to One-way ANOVA with post-hoc Tukey test.

CKs regulate vascular development and are required for the formation of the vascular cambium [58,59,60]. CKs have been found in the xylem and phloem sap of various plant species [61]. In *P. major*, we detected different CK forms in both mesophyll and vascular tissue (Figure 5). In general, CKs accumulated predominantly in the vascular tissue supporting the role of CK for the growth and development of this tissue. We detected the precursor and transport forms isopentenyladenine riboside (IPR) and *cis*-zeatin riboside (CZR), but not *trans*-zeatin riboside (TZR) under control B (2.4 mg B (kg soil substrate)^−1^). The sum of zeatin (Z)-related precursors was about six to ten times higher than that of IP-precursor and bioactive forms. This ratio is in line with an earlier report of CK levels in *P. major*, where CKs were measured in whole shoots or roots and quantified using a soybean callus bioassay [22]. The data suggests that CK are efficiently loaded into the phloem or xylem in the shoot or root, respectively, and that their metabolism, i.e., activation of precursors and inactivation by glycosylation also occurs predominantly in the vasculature, since mesophyll concentrations of all forms were significantly lower than those of the vasculature (Figure 5).

Interestingly, the concentrations of IPR, CZR, and TZR were strongly and mostly specifically elevated in the vasculature under the low B condition (0.1 mg B (kg soil substrate)^−1^). The active forms *cis*-zeatin and *trans*-zeatin were below the detection limit of 0.1 ng g^−1^ DW in all tissues and conditions. The only highly bioactive form detected was isopentenyladenine (IP), which increased in the vasculature of plants grown under the low B condition.

Transport of IP-type CK occurs primarily in the phloem and that of TZ and CZ-ribosides primarily in the xylem [60,62,63]. Because of this compartmentalization of the different CK forms, it has been suggested that Z- and ZR-type CKs signal stress or nutrient deficiencies perceived by the root via the xylem to the shoot and IP-type CKs transduce signals in a systemic manner via the phloem [62]. Although our data does not discriminate between phloem and xylem, they are in line with such a scenario. The CK concentrations measured in the *P. major* vascular bundle fraction most likely reflect concentrations of IP-derived or CZ-derived CK in the phloem and xylem, respectively (Figure 5). It is likely that also perception and response of nutrient limitations, in this case, B limitation, is transmitted to different plant organs and tissues via vascular CK broadcast. The B limitation-dependent increase of IP- and CZ-forms is opposed to results obtained in rapeseed where concentrations of CZR, CZ, IPR, and IP increased in a shoot B content-dependent manner [52]. However, in contrast to rapeseed, the low soil B conditions had no visible impact on the vegetative growth of *P. major* (Figure 1) and increased CK levels may signal a continuation of growth despite suboptimal B levels here.

#### 2.4.2. Brassinosteroids (BRs)

**Figure 6:**
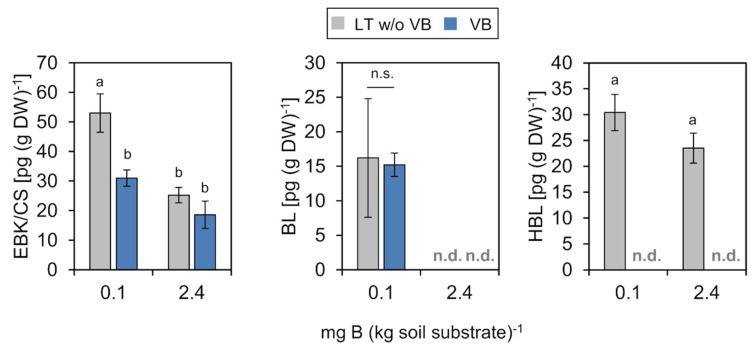
Concentrations of brassinosteroids in vascular bundles (VB) or leaf tissue without VB (LT w/o VB) of *P. major* plants grown on low B (0.1 mg B (kg soil substrate) ^−1^) or B-sufficient (2.4 mg B (kg soil substrate)^−1^) soil substrate. EBK/CS (epicastasterone/castasterone) and HBL (homobrassinolide) accumulate in non-vascular tissue. CS and BL (brassinolide) concentrations increased in both tissues of B-deficient plants. Bars represent averages from three different measurements ± SD. n.d.= not detected. Error bar labels with different letters indicate significant differences at *p* < 0.05 between treatments and tissues according to One-way ANOVA with post-hoc Tukey test. n.s. = no significant differences (*p* > 0.05).

BRs and castasterone (CS) in particular promote growth mainly by enhancing cell elongation [64,65] and plants with reduced BR biosynthesis exhibit dwarf phenotypes [66]. In *P. major*, CS concentrations were similar in vascular bundles and mesophyll tissue under normal soil B conditions, but increased about 2-fold in the mesophyll of plants grown at low B in comparison to plants grown on B-sufficient soil substrate (Figure 6). Only a weak increase in CS concentration was observed in vascular bundles. Brassinolide (BL) was not detected in tissues from plants grown at B-sufficient conditions, but accumulated in bundles and mesophyll of plants grown at low B. A third BR form, homobrassinolide (HBL) was only detectable in the mesophyll and not in the vascular bundle fraction. HBL concentrations in this tissue did not increase significantly at low B (Figure 6). However, in addition to CS, BL and HBL have also been reported to support plant growth and photosynthetic efficiency of leaves under stress conditions. In mung bean (*Vigna radiata* (L.) Wilczek) for instance, toxic effects of excess B could be alleviated by the exogenous supply of 28-HBL by stimulating antioxidative enzyme activity [67].

We speculate that the elevated BR levels in the mesophyll of *P. major* contributed to the ability of this species to maintain vegetative growth and cell expansion under suboptimal B conditions. This assumption is based on observations that in species which are sensitive to B deficiency, e.g., in rapeseed or sunflower, (i) B deficiency leads to a failure of cell elongation, a hyperproliferation of cells, and a stunted growth [2,7], all phenotypes which resemble that of plant mutants which are de-regulated in their BR metabolism and (ii) that CS levels are upregulated in a B-dependent manner [52], together indicating that leaf CS concentrations might be too low to promote cell expansion under B-deficient conditions in B deficiency sensitive plant species. A possible causative link between BR levels and B deficiency tolerance has to be tested in future, e.g., by growing transgenic *A. thaliana* plants with enhanced BR biosynthesis on limiting B conditions.

#### 2.4.3. Salicylic Acid (SA)

**Figure 7:**
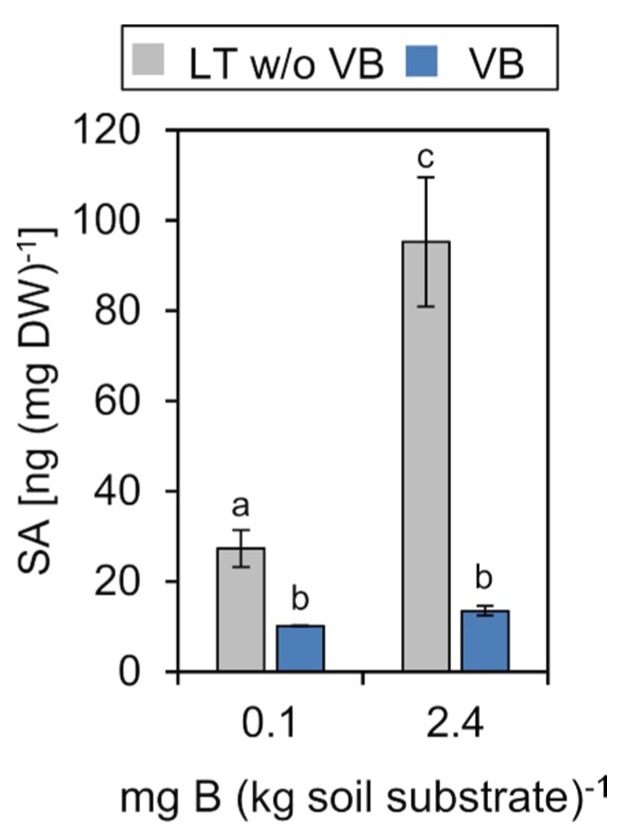
The concentration of salicylic acid (SA) in vascular bundles (VB) or leaf tissue without VB (LT w/o VB) of *P. major* plants grown on low B (0.1 mg B (kg soil substrate) ^−1^) or B-sufficient (2.4 mg B (kg soil substrate) ^−1^) soil substrate. Error bar labels with different letters indicate significant differences at *p* < 0.05 between treatments and tissues according to One-way ANOVA with post-hoc Tukey test.

SA levels decreased to about one fifth in the mesophyll tissue of plants grown under low B when compared to the B-sufficient soil condition. Notably, SA levels were markedly lower in vascular tissue under both soil B conditions in comparison to the mesophyll (Figure 7). The pronounced decrease of SA in the mesophyll at low B, however, was not accompanied by a similar decrease in the vasculature. In many plant species, the main SA biosynthetic route occurs via chorismate/isochorismate in the chloroplasts [68,69]. The vasculature does not include large amounts of photosynthetically active cells containing chloroplasts. SA biosynthesis and accumulation in the VB may be, therefore, limited to a small set of plastid-carrying parenchyma and phloem companion cells. This can explain why SA levels were low in VB, but high in mesophyll under the normal B condition (Figure 7). Studies addressing systemic acquired resistance signaling demonstrated that SA is involved in the transmission of biotic stress responses via the phloem, but did not represent the phloem mobile factor itself [70,71]. More recent studies showed that the methylated derivative 6-methylsalicylic acid (MeSA) had a higher membrane permeability in comparison to SA and indicated that MeSA was phloem mobile [72,73]. In respect to these results, it is tempting to speculate that the here observed reduction of SA under low B in the mesophyll was followed by a concomitant increase in MeSA in the vasculature. It will be highly informative in future to analyze vascular tissue levels of MeSA under different nutrient deficiencies, including that of B to further elucidate a potential role of SA/MeSA in long-distance nutrient-deficiency signaling.

#### 2.4.4. Abscisic Acid (ABA)

ABA is a known stress hormone and an inhibitor of leaf expansion. In our B-dependent growth analysis of *P. major*, levels of ABA, its glucosyl-ester storage form (ABA Gluc), and the catabolic inactivation products dihydrophaseic acid (DHPA) and phaseic acid (PA) were all decreased in B deficiency conditions. This is in contrast to reports from rapeseed where plants, which grow under B deprivation, possessed higher ABA contents compared to plants grown under B-sufficient conditions [10,52].

**Figure 8:**
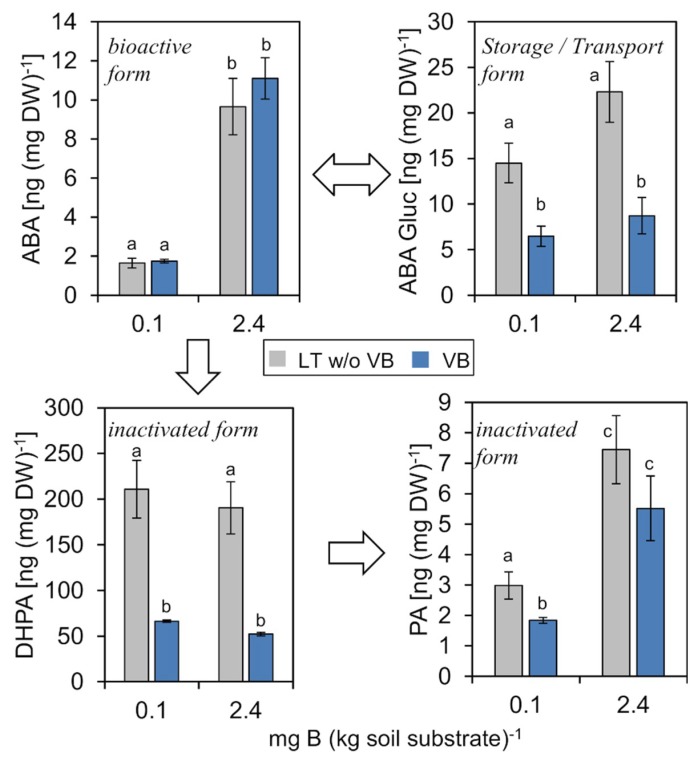
Concentration of abscisic acid (ABA), ABA-glucose ester (ABA Gluc), dihydrophaseic acid (DHPA), and phasic acid (PA) in vascular bundles (VB) or leaf tissue without VB (LT w/o VB) of *P. major* plants grown on low B (0.1 mg B (kg soil substrate)^−1^) or B-sufficient (2.4 mg B (kg soil substrate)^−1^) soil substrate. Arrows indicate metabolic conversions. Error bar labels with different letters indicate significant differences at *p* < 0.05 between treatments and tissues according to One-way ANOVA with post-hoc Tukey test.

Interestingly, ABA concentrations under low B supply were considerably lower in both the vasculature and the mesophyll fraction in comparison to sufficient B supply. These data indicated that *P. major* plants did not respond with an inhibited growth or cell division to the low B condition as was also apparent from the comparable shoot dry weights of plants grown under the different conditions (Figure 8). The decrease of ABA, a repressor of leaf growth, and the simultaneous increase of BRs (Figure 6), which stimulate cell and leaf expansion, suggest that *P. major* actively adjust levels of growth-related PHs against an otherwise possible B-deficiency induced growth arrest. The decrease of also the inactivation products DHPA and PA indicates that not ABA degradation, but de novo ABA synthesis was greatly reduced under the low B condition.

## 3. Materials and Methods

### 3.1. Growth Conditions and Plant Tissue

*P. major* plants used in the analyses were of an inbred line used during prior studies [20,23]. Plants were grown in greenhouse conditions under a long-day regime for six weeks in a soil substrate with defined B conditions [7]. Preparation of soil substrate with defined B conditions was prepared, as described in Reference [7]. Vascular strands were manually separated from leaf tissue as previously described [13,20]. Isolated bundles and remaining leaf tissue from which the bundles had been pulled out were immediately frozen in liquid nitrogen, freeze-dried and kept at −80 °C until further extraction and analysis of metabolites.

### 3.2. PAM Measurements

Photosynthetic activity was measured with pulse amplitude modulated (PAM) fluorometry. An Imaging-PAM *M-Series*-System (Heinz Walz, Effeltrich, Germany) was used. Plants were placed in the dark for 12 min to deplete the energy of PSII. Afterwards, the capacity of PSII was measured by saturating it with a series of PAR 76 (µmol photons m^−2^ s^−1^) light-pulses. Recorded fluorescence was used for calculation of the effective quantum yield of PSII [*Y(II) = (Fm’-F)/Fm’*], quantum yield of regulated energy dissipation [*Y(NPQ) = 1 - Y(II) - 1/(NPQ + 1 + qL(Fm/Fo-1))*] and of non-regulated energy dissipation [*Y(NO) = 1/(NPQ + 1 + qL(Fm/Fo-1))*]. Required factors were calculated by the formulas [*NPQ = (Fm-Fm’)/Fm’*], [*qN = (Fm-Fm’)/(Fm-Fo’)*], [*Fo’ = Fo/ (Fv/Fm + Fo/Fm’)*], [*qP = (Fm’-F)/(Fm’-Fo’)*] and [*qL = (Fm’-F)/(Fm’-Fo’) x Fo’/F = qP x Fo’/F*].

### 3.3. Analyses of Plant Hormones

Comprehensive protocols of the plant hormone extraction and analysis can be found in Reference [52]. The extraction and analysis of the plant hormones in this study were performed, as described in detail in Method S1 of the supporting information section in Reference [52]. Deviating from this protocol, isolated vascular tissue (=VB) and enriched mesophyll (=LT w/o VB) were harvested and pooled from 3 × 6 plants per treatment. The tissues were immediately collected in liquid nitrogen, freeze-dried, and ground to a fine powder using a mixer mill (Retsch, Haan, Germany). For analysis of ABA, SA, CK, and BR about 80 mg of dry matter were transferred to 2 mL Eppendorf tubes containing 750 µl ice-cold 0.1% formic acid solution, sonicated for 30 s, and extracted at 4 °C for 15 min using an overhead shaker. Samples were centrifuged at 4 °C and 16,000× *g*. Supernatants were transferred to new tubes, and the pellet was re-extracted. Supernatants were combined and further used for ABA/SA/CK analysis, as described in Reference [52]. For brassinosteroid extraction, the pellets collected from the ABA/SA/CK extraction were used. The pellets were treated with 750 µl 50% acetonitrile and extracted, as described in Reference [52]. 

### 3.4. Analyses of Sugars

Vascular bundles and remaining leave tissue were separately harvested and pooled from 6 × 6 plants, immediately transferred to liquid nitrogen and freeze-dried. Tissues were ground to a fine powder using a mortar and pistil. About 50 mg of ground material was extracted with 1 mL 80% EtOH at 80 °C for 1 h in a 2 mL Eppendorf tube. Samples were centrifuged for 10 min at 11,000× *g* and the supernatant collected. The pellet was extracted again, as described above. Supernatants were combined and evaporated in a vacuum concentrator (Eppendorf, Hamburg, Germany) and pellets resolved in 500 µl sterile water. Quantification of glucose, fructose, and sucrose was performed using an NADP-coupled enzymatic test [74]. Briefly, 20 µl sample were mixed with 190 µl premix (100 mM HEPES pH 5.7, 10 mM MgCl_2_, 2 mM ATP, 0.8 mM NAD, 5 U Glc-6-P-Dehydrogenase). Formation of NADH was measured at 340 nm in a plate reader (Tecan, Männedorf, Switzerland). A340 was measured for 20 min (blank), 40 min after addition of 1.5 U hexokinase (for glucose), 30 min after addition of 1 U phosphogluco-isomerase (for fructose), and 40 min after addition of 1 U invertase (for sucrose). Sugar concentrations were calculated based on NADH concentrations according to the law of Lambert-Beer. Sorbitol was analyzed with an ICS-3000 ion chromatography system (Dionex, Sunnyvale, CA, USA) equipped with a Carbo Pac MA1 column. The measurements were performed, as described previously [20]. 

### 3.5. In planta Esculin Trafficking

Eight-week-old *P. major* plants grown at 20 °C under short day conditions (10 h light, 14 h darkness) on low B or B sufficient conditions were used for the analysis. One source leaf per plant (usually from leaf stage 3 to 4) was roughened at the adaxial side with fine sandpaper (grade 1000). About 1 mL of a 100 mM esculin sesquihydrate (Carl Roth, Karlsruhe, Germany) solution were distributed over the injured leaf surface with a plastic pipette. Ten plants per B supply condition were treated. Treated leaves were coated with plastic foil and kept at short days at 20 °C. The esculin-loaded source leaves were detached, and hand sections of petioles (cut with a sharp razor blade) were analyzed for esculin fluorescence with a Leica TCS SP5II confocal microscope (Leica, Mannheim, Germany) using an HCX PL APO lamda blue 20.0 × 0.70 IMM UV objective. The emission bandwidths were 440–465 nm for detection of esculin fluorescence and 594–631 nm for lignin fluorescence.

### 3.6. ICP-MS-Based Analyses of Elements

Vascular bundles and remaining leave tissue were separately harvested from 5 × 6 plants per B supply condition, freeze-dried, and ground to a fine powder. About 50 mg per tissue sample and replicate were used for the ionome analysis. The analysis was performed using high-resolution (HR)-ICP-MS, as described in full detail in Reference [75].

### 3.7. Statistical Analysis

Principal Component (PC) and correlation analysis were performed using Clustvis [76]. Prior to PCA, values were ln(x + 1) transformed and Pareto scaling was applied. 

Data were checked for normal distribution by using an online version of the Shapiro-Wilk normality test (http://sdittami.altervista.org/shapirotest/ShapiroTest.html). Data were considered to be distributed normally when the null hypothesis was not rejected with a threshold of *p* = 0.05 (data in Figure 1, Figure 2, and Figure 4). Normally distributed data were used for calculation of significant differences between treatments. Because of sample sizes < 5, no normality test was conducted for plant hormone data. Significant differences were calculated by performing double-sided *t*-test or one-way ANOVA with post-hoc Tukey HSD test (*p* < 0.05) [77] as indicated in the corresponding figure legends.

## 4. Conclusions

The vascular tissue has a primary function in long-distance transport of water, nutrients, and photo-assimilates. It also transfers developmental triggers in response to environmental stimuli to recipient tissues or organs by distributing signaling molecules and plant hormones, and, therefore, acts as an information superhighway within the plant. Boron deficiency symptoms are diverse and lead to developmental and functional impairments of the affected tissues. In *P. major*, B deficiency affected generative, but not vegetative growth. Our results showed that different plant hormones accumulate differentially between the leaf vasculature and the leaf mesophyll and respond tissue-specifically to low B availability. In particular, we observed accumulation of cytokinins in the vasculature and of brassinosteroids in the mesophyll under low B availability. These hormones might trigger biochemical and physiological responses of *P. major* plants allowing this species to maintain growth under B conditions that would otherwise be fatal for other dicotyledonous species. In future, it will be highly interesting to conduct proof-of-concept studies and rescue experiments of B-inefficient plant species and varieties using cytokinin and/or brassinosteroid species or inhibitor application experiments.

## Supplementary Materials

Supplementary materials can be found at https://www.mdpi.com/1422-0067/20/16/3882/s1. Supplementary Figure S1: Information about PCA loadings and explained variances of PCs

## Abbreviations

ICP-MS	Inductively coupled plasma-mass spectrometry
LT w/o VB	Leaf tissue without vascular bundles = mesophyll
PH	Plant hormone
VB	Vascular bundle(s)
