# Supplementary material for: Boron Deficiency Effects on Sugar, Ionome, and Phytohormone Profiles of Vascular and Non-Vascular Leaf Tissues of Common Plantain (Plantago major L.)
Source: Int J Mol Sci. 2019 Aug 9;20(16):3882. doi: 10.3390/ijms20163882 (PMC6719229; doi:10.3390/ijms20163882)
Supplement: Supplementary file 1 [file ijms-20-03882-s001.pdf]

A

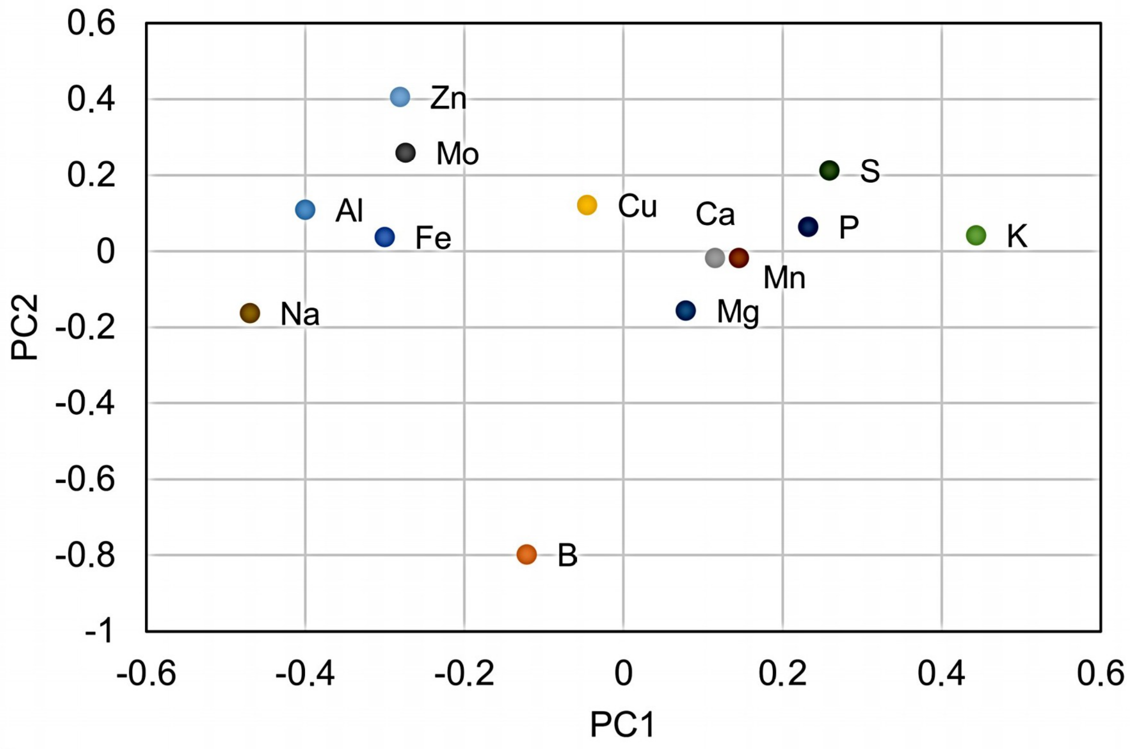

B

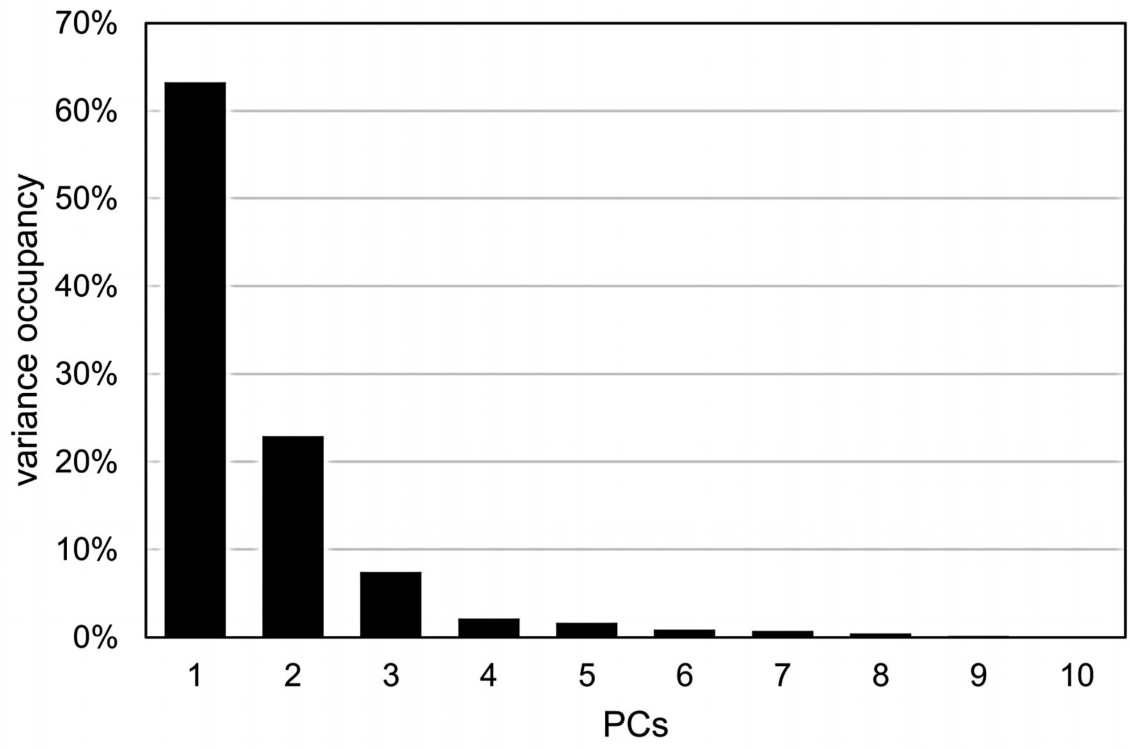

Supplementary Figure S1: Information about PCA loadings and explained variances of PCs for the PC analysis of elemental distribution**. A)** PCA loadings (variables). **B)** explained variances of the first 10 principal components (PCs)
